# Detection of HIV cDNA Point Mutations with Rolling-Circle Amplification Arrays

**DOI:** 10.3390/molecules15020619

**Published:** 2010-01-27

**Authors:** Lingwei Wu, Quanjun Liu, Zhongwei Wu, Zuhong Lu

**Affiliations:** 1 State Key Laboratory of Bioelectronics, Southeast University, Nanjing, 210096, China; E-Mail: lwwu@ncu.edu.cn (L.W.); 2 Jiangxi-OAI Joint Research Institute, Nanchang University, Nanchang, 330047, China

**Keywords:** point mutation, mismatch, rolling-circle amplification, T4 DNA ligase

## Abstract

In this paper we describe an isothermal rolling-circle amplification (RCA) protocol to detect gene point mutations on chips. The method is based on an allele-specific oligonucleotide circularization mediated by a special DNA ligase. The probe is circularized when perfect complementary sequences between the probe oligonucleotide and HIV cDNA gene. Mismatches around the ligation site can prevent probe circularization. The circularized probe (C-probe) can be amplified by rolling circle amplification to generate multimeric singlestranded DNA (ssDNA) under isothermal conditions. There are four sequence regions to bind respectively with fluorescent probe, RCA primer, solid probe and HIV cDNA template in the C-probe which we designed. These ssDNA products are hybridized with fluorescent probes and solid probes which are immobilized on a glass slide composing a regular microarray pattern. The fluorescence signals can be monitored by a scanner in the presence of HIV cDNA templates, whereas the probes cannot be circularized and signal of fluorescence cannot be found. The RCA array has capability of high-throughput detection of the point mutation and the single-nucleotide polymorphism (SNP). The development of C-probe-based technologies offers a promising prospect for situ detection, microarray, molecular diagnosis, single nucleotide polymorphism, and whole genome amplification.

## 1. Introduction

With completion of the sequencing of the human genome [1], new genes are being discovered at an accelerated pace as well as determination of the function of these genes and potential associations of these genes and mutations within them to particular phenotypes. The whole human genome seems to encode 20,000–25,000 protein-coding genes, and certain mutations will lead to dysfunctional proteins which may give rise to diseases. Therefore, to find some new method of mutation assay which can simultaneously identify all these types of variations is most desirable. Mutation detection is usually focused on very specific sequencing, or mini-sequencing, of a particular gene locus, such as a single-nucleotide polymorphism (SNP). Oligonucleotide probes provide a useful tool for the detection of target nucleic acids through the formation of a double-helical structure between complementary sequences. However, hydrogen bonds between target and probe hybrids are inherently weaker than covalent bonds, thus, making it more susceptible to breakage during stringent washes. To overcome this problem, Nilsson *et al*. [2] synthesized a circularizable oligonucleotide probe (C-probe) for *in situ* target localization and detection. On a target DNA, the two ends of the probe become juxtaposed and can be joined by DNA ligase. The C-probe was introduced to detect a mutation in a target because even a single nucleotide change in the target prevents ligation. The utility of circularizable oligonucleotides probe had been demonstrated for the detection of target nucleic acid sequences. This approach shows greater sensitivity than conventional PCR [3,4]. Several methods [5,6,7,8,9,10] have been designed to improve the detection sensitivity by amplifying the closed C-probe. The initial strategy was introduced to amplify the closed C-probe by the rolling circle amplification (RCA) mechanism as observed in *in vivo* bacteriophage replication [7]. This type of amplification, however, only results in linear growth of the products with several thousand-fold amplifications.

**Figure 1:**
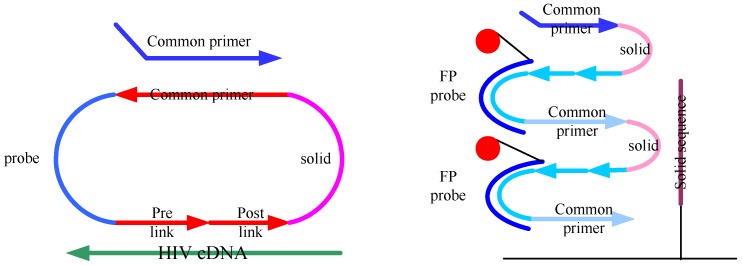
The RCA detection gene point mutation strategy: probe circularization by DNA ligase, and rolling circle amplification by phi29 DNA polymerase. The circularizable oligonucleotides probe was comprised of four regions: primer region, solid region, template region and probe region. The circularizable probe was initially introduced to detect a mutation in a target and closed the nick by incubating with a DNA ligase. Then binding complementary common primer, the circularized probe (C-probe) was amplified by phi29 DNA polymerase to generate multimeric single stranded DNA (ssDNA). The amplification products were hybridized with FPs(fluorescent probes) and solid probes that were immobilize to the surface of glass slide, and the fluorescent signals can be detected by scanner.

Microarray-based analyses have been well established and are currently used in a wide range of biological assays [11,12]. A DNA-chip based on streptavidin-coated microwells was used to detect single-nucleotide polymorphisms using molecular DNA switches and isothermal rolling-circle amplification. In this paper we describe a novel protocol for mutation analysis based on glass-chip or lab-in-a-tube (Figure 1). The circularizable oligonucleotides probe was comprised of four regions: primer region, solid region, template region and probe region. A single forward primer complementary to the linker region of the C-probe and a phi29 DNA polymerase with strand displacement activity are employed. The polymerase extends the bound primer along the closed C-probe for many revolutions and displaces upstream sequences, producing a long singlestranded DNA (ssDNA). The amplification products hybridized with fluorescent probes and solid probes that were immobilized on the surface of glass slide. The fluorescent signals were detected by scanning. The DNA arrays have a great potential to provide a fresh and attractive scheme that has the ability of high-throughput detection of the point mutation and exclusion of electrophoresis. Using this new approach we successfully monitored point mutations of HIV cDNA.

## 2. Results and Discussion

### 2.1. Probe circularization and RCA reaction

**Figure 2:**
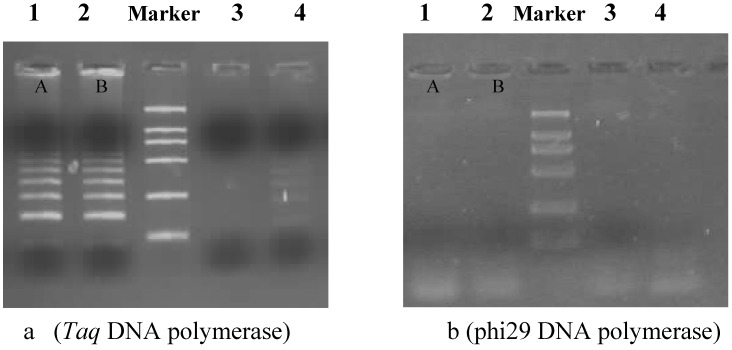
Electrophoresis analysis of RCA product on 1.2% agarose gel. 20 pmol circularizable probe and 20 pmol DNA template were used at RCA reaction with Φ29 DNA polymerase (b): (1) OCPA-TA, (2) OCPA-TA, (3) OCPA-TB, (4) OCPA-TB. The RCA products (A and B) were shown to detect their corresponding artificial templates, but at (3) and (4) no RCA product signal was detected. phi29 DNA polymerase was replaced by *Taq* DNA polymerase as contrast (a).

Two circularizable probes were designed to target various two artificial templates (Table 1). Between the two artificial templates (TA and TB), there is only a one base difference (with an G/C wild type/mutation type mismatch) in order to detect the point mutation. Initial testing of the two probes and their corresponding artificial templates indicated that probe readily bound to the corresponding template and the RCA product signals could be easily detected. Electrophoretic analysis of RCA amplification products A and B is shown in Figure 2. However, when artificial template TB was used as nonspecific template hybridized with OCPA (in Table 1) for specificity tests, no signals were observed, indicating highly specific binding for probes (Figure 2). The availability of ligases that accurately distinguish DNA sequences has made by oligonucleotide ligation assays. Circularizable probes are also suitable to distinguish single nucleotide sequence variants in complex genomes. T4 DNA ligases have been isolated from a number of organisms and characterized for their ability to discriminate between matched and mismatched substrates. RCA reaction was performed by using phi29 DNA polymerase with strand displacing activity. The phi29 DNA polymerase can dissociate the probe–target hybrid and replicate the circularized probe once or twice also when the probe remains linked to a circular target strand. The phi29 produces have high molecular weight material that hardly enters the gel. The fragments in the loading well were multimers containing single-stranded loops. The *Taq* polymerase, by contrast, was used to replace phi29 DNA polymerase. Primer CP is the same for both reactions, and primer RCP is as allele-specific reverse complemented primer, binding to unique sequences in the complementary strands of the circular probe (Figure 2a). With Taq polymerase, the RCA reaction produced many bands of different sizes from ~70 bps to the loading well. Production of the bands depended on the presence of the primers, the template and DNA polymerase. The slow migrating DNA and the DNA in the loading well were replicated intermediates containing single-stranded loops and double stranded loops. This difference could be due to the fact that the two polymerases differ in their process and their capacity for strand displacement.

### 2.2. Hybridization and DNA microarray

**Figure 3:**
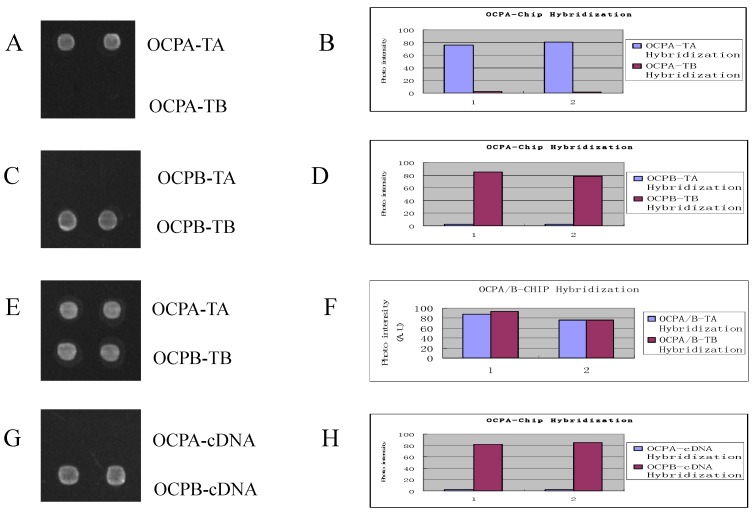
Images and signal intensity plot of the hybridization of RCA products to the DNA array. There are two sorts of probes: SSA (upper) and SSB (lower) on the chip. A and B showed the result of hybridization of OCPA-TA and OCPA-TB RCA products with chip probes; C and D showed the result of hybridization of OCPB-TA and OCPB-TB RCA products with chip probes; E and F showed the result of hybridization of mix of OCPA-TA and OCPB-TB RCA products with chip probes. G and H showed the result of the hybridization of OCPA-cDNA and OCPB-cDNA with chip probes.

The chips were spotted with four dots: two up dots were spotted with solid sequence probes SSA and the down were solid sequence probes SSB. RCA amplification products A and B were hybridized with fluorescent probes (FPs) and solid sequence probes respectively, and the scan results were showed on Figure 3. The RCA products of OCPA only hybridized with SSA and fluorescent probe, and the products of OCPB with SSB and FB. Furthermore, the mix of products of OCPA and OCPB can hybridize respectively with SSA or SSB. The method employs two C-probes to confer highly accurate mutation discrimination and high-throughput detection. Using this new approach we successfully monitored point mutations from HIV cDNA (G, H in Figure 3). The HIV cDNA comes from our lab. Its sequence references NCBI GeneBank (ref|NC_001802.1| Human immunodeficiency virus 1, gb|AF033819.3| HIV-1, complete genome, Sbjct 2664). Some middle sequence of the template TB is same as HIV cDNA, so the template TB was replaced by HIV cDNA. 

## 3. Experimental

### 3.1. Materials and Apparatus 

The special phi29 DNA polymerase and polymerase chain reaction (PCR) reagents were obtained from TakaRa Company. T4 DNA ligase was purchased from New England Biolabs. 3`-Amino-modified fluorescence probes and other oligonucleotides used in this study were synthesized by Shanghai Invitrogen Inc. (China). 3-Aminopropyltriethoxysilane (APTES, 99%) was purchased from Aldrich. Microscope slides (DAKO, Catalog no.S3003) were used as PCR reaction containers. The instruments used in this experiment were: PCR thermocycler (PTC-200, MJ Research), real-time PCR (Peltier thermal cycler, Eastwin), microarray scanner (LS-300, Tecan), and microarray spotter (PRXSYS 5500, Cartesian). 

### 3.2. Circularizable probe design

The oligonucleotide probes used in these experiments were designed especially with 80 to 90 nucleotides (nt) in length, consisting of four adjacent target complementary sequences (15 to 20 nt), with a region to facilitate the loop formation, a region to provide a template for RCA primer binding, a region to bind the solid sequence and the region for fluorescent probe binding. The sequence region to facilitate the loop formation was parted, which could be ligated with T4 DNA ligase according to the target complementary sequences. To demonstrate a working molecular model and to allow optimization of assay conditions, artificial DNA templates were synthesized. The oligonucleotides used in this study were synthesized and purified by Invitrogen Inc. (Shanghai, China). These (NN…) sequences at the 5' and/or 3' termini of the artificial templates and CP showed the sequence may be replaced with any other sequences. Their sequences are shown in Table 1.

### 3.3. Probe Circularization

The probe specifically binds the target template and becomes circularized by DNA ligase. Probe OCPA (20 pmol) was hybridized with equimolar ratios of artificial template TA and circularized by incubation with 20 U of T4 DNA ligase in 1×reaction buffer in a 20 μL reaction volume. The cocktail was incubated at 16 °C for 30 min, and the reaction was terminated by heating the samples to 75 °C for 15 min, so did OCPB and TB. Separate control reactions were also performed with open circle probe A (OCPA) and artificial template B (TB). No ligation reactions were also made by omitting ligase from the above reaction.

### 3.4. Molecular model of RCA in the liquid phase

After ligation and circularization of the probe, amplification of the circular probe by RCA was performed by using *Φ29* DNA polymerase with strand displacing activity. RCA reactions were performed in a 50 μL volume containing *Φ29* DNA polymerase (20 U), 400 μM deoxynucleoside triphosphate mix, 40 pmol of each RCA primer (CP), and 5 μL of the original ligation mix. Circularized probe signals were amplified by incubation at 37 °C for 90 min and visualized on a 1.2% agarose gel under UV illumination. The *Taq* polymerase, by contrast, was used to replace phi29 DNA polymerase. Primer CP is the same for both reactions, and primer RCP is as allele-specific reverse complementary primer, binding to the circular probe. The RCA cocktail contained *Taq* DNA polymerase (10 U), 400 μM deoxynucleoside triphosphate mix, 40 pmol of each RCA primer (CP), 10 pmol RCP, and 5 μL of the original ligation mix. The RCA cocktail was heated at 95 °C for 5 min, followed by incubation at 65 °C for 30 min and heating at 90 °C for 10 min to terminate the reaction.

### 3.5. Manufacture of DNA-chip *[13]*

The amino-silane derived glass slides were cleaned with deionized distilled water and incubated in 5% glutaraldehyde in 0.1M phosphate buffered saline (PBS) buffer (pH 7.4) for 2 h. Then the slides were thoroughly washed twice with methanol, acetone and deionized distilled water, and dried. Spotting solutions were obtained by dissolving probes (SSA or SSB) in sodium carbonate buffer (0.1M, pH 9.0) at the concentration of 10 mM Pin-based spotting robot PixSys5500 (Cartesian Tech. Inc.) with SMP3 pin was used to perform probe array spotting. After spotting, glutaraldehyde derived glass slides were incubated at room temperature for 2 h and at 37 °C for 2 h. Next, the slides were soaked twice in 0.1% SDS for 2 min at room temperature with vigorous agitation and then transferred the slides into a sodium borohydride solution [made by dissolving 1.5 g NaBH_4_ (Sigma) in 450 mL PBS, and then adding 133 mL of 100% ethanol] for 5 min at room temperature to reduce free aldehydes. Finally, the slides were washed thoroughly in 0.1% Tween, rinsed in distilled water and dried by a flow of nitrogen gas. The probe array can be used for immediately hybridization on a solid surface or stored at 4 °C for future use.

### 3.6. Hybridization on solid surface and detection

As for the heterogeneous nuclease PCR assay, glass slides on which 5’ amino-modified probes were covalently bonded, were subjected to a 1 h blocking step using 5 × saline sodium citrate (SSC) buffer containing 0.1% Tween-20, 0.1% BSA (Bovine Serum Albumin), washed in 5 × SSC buffer and finally washed in water 30 μL. RCA amplification products combining with 10μL Hybridization buffer (containing 35% formamide, 0.5% SDS (Sodium dodecyl sulfate), 2.5 × Denhardt’s and 4 × saline sodium phosphate EDTA (SSPE) and 10 μL fluorescent probe (10 μmol/L) were incubated in PCR tube. 10 μL mix were spotted on above glass slides. The microarray hybridization was conducted in a moist hybridization chamber under a cover slip at 42 °C for 2 h. After hybridization, the slide was rinsed and washed at room temperature with 6 × SSC- 0.1% SDS buffer for 1 min, 6 × SSC buffer for 1 min, 2 × SSC and dried by a flow of nitrogen gas. The DNA-chip was completed and could be scanned with the Tecan LS-300.

## 4. Conclusions

The use of circularizable probes on DNA chips can greatly enhance sequence identification with single-nucleotide resolution. The RCA array has the ability of high-throughput detection of the point mutation and exclusion electrophoresis, and was suitable to distinguish single nucleotide sequence variants in complex genomes such as human genomes.

## Figures and Tables

**Table 1:** Synthesized oligonucleotide sequences for experiment.

Probes and Primers	Sequences
OCPA—Open circle probe A	5`p-GCTACATACAAATCGCACTCGACTTGCTGATACGGCATAGTCGTACCGACCTGTAGGCTGAGAACGGGAAGCTGTGCTAAGTCAGAT-3
OCPB— Open circle probe B	5`p-CCTACATACAAATCGCACTCGACTTGCTGATACGGCATAGTCGTACCGACCTGTACGTGCTGGTGTGCATGCCTGTGCTAAGTCAGAT-3`
TA— Artificial template A	5`-GATTTGTATGTAGCATCTGACTTAGCACAG-3`
TB—Artificial template B	5`-GATTTGTATGTAGGATCTGACTTAGCACAG-3
CP—Common primer	5`-TACAGGTCGGTACGACTATGC-3`
RCP—primer	5`-GCATAGTCGTACCGACCTGTA-3`
SSA—Solid sequence A on glass chip	5`-NH_2_-TTTTTTTTTTTTTTTTTTTTGGCTGAGAACGGGAAG-3`
SSB—Solid sequence B on glass chip	5`-NH2-TTTTTTTTTTTTTTTTTTTTCGTGCTGGTGTGCATGC-3`
FP— fluorescent probe	5`-GCACTCGACTTGCTGATACGTTTT-cy3-3`
